# In-hospital mortality predictors among COVID-19 patients in the West Bank, Palestine: a multi-center retrospective cohort study

**DOI:** 10.3389/fmed.2025.1634917

**Published:** 2025-10-08

**Authors:** Hamzeh Al Zabadi, Danyah Khalid, Ibrahim Taha, Mohammed Rabae, Jamal Qaddumi

**Affiliations:** ^1^Master of Public Health Management, Faculty of Graduate Studies, An-Najah National University, Nablus, Palestine; ^2^Public Health Department, Faculty of Medicine and Health Sciences, An-Najah National University, Nablus, Palestine; ^3^Optometry Department, Arab American University, Ramallah, Palestine; ^4^Dura Governmental Hospital, Palestinian Ministry of Health, Hebron, Palestine; ^5^Department of Nursing, Faculty of Medicine and Health Sciences, An-Najah National University, Nablus, Palestine

**Keywords:** COVID-19, in-hospital mortality, Palestine, predictors, cox regression model

## Abstract

**Background:**

COVID-19 pandemic has presented unprecedented challenges to global healthcare systems, particularly in resource-limited settings like Palestine. Identifying clinical, demographic, and laboratory predictors of in-hospital mortality is crucial for improving outcomes, guiding treatment, and optimizing resource allocation.

**Objectives:**

This study aimed to determine the key demographic, clinical, laboratory, and treatment-related factors associated with in-hospital mortality among COVID-19 patients admitted to six governmental hospitals across the West Bank, Palestine.

**Methods:**

A retrospective cohort design was employed using data from 200 confirmed COVID-19 patients hospitalized between November 1, 2020, and February 1, 2021. Bivariate analyses were conducted using Chi-square and *t-*tests. Statistically significant variables (*p* < 0.05) were entered into logistic and Cox regression models to identify independent predictors of mortality and time to death.

**Results:**

The overall In-hospital mortality rate was 43%. Obesity was the strongest predictor of mortality (OR = 5.73, *p* < 0.001; HR = 2.66, *p* < 0.001). Pregnant women showed alarmingly high mortality (81%), though this did not remain significant in multivariate analysis due to small sample size. Widowed patients were significantly more likely to die (HR = 2.77, *p* = 0.019), emphasizing the role of social support. Hospital type was another independent predictor, with patients at Dura Hospital experiencing higher odds of death (OR = 3.26, *p* = 0.017). Key laboratory findings included significantly lower PaO_2_, serum creatinine, and random blood sugar levels among non-survivors. Actemra (tocilizumab) use was associated with significantly lower mortality (0% vs. 46%, *p* = 0.001), while vancomycin and ceftriaxone were associated with higher mortality, likely reflecting use in severe cases.

**Conclusions:**

This study identified key predictors of COVID-19 mortality in Palestine, emphasizing the need to prioritize high-risk groups such as obese and pregnant patients. The findings highlight disparities between hospitals and reinforce the importance of standardizing care across healthcare facilities. These insights can help shape evidence-based health policies tailored to the Palestinian context and could serve as a foundational dataset for preparedness in future pandemics or resurgences of COVID-19.

## Introduction

The global outbreak of coronavirus disease 2019 (COVID-19) has resulted in significant morbidity and mortality, particularly among hospitalized populations. As of early 2025, the virus has caused over 7 million deaths worldwide, World Health Organization (38). In Palestine, the West Bank has experienced several intense waves of infection, placing immense pressure on the healthcare system. Understanding the predictors of in-hospital mortality is essential for improving patient management and optimizing the use of limited healthcare resources (1).

Recent studies consistently show that advanced age remains one of the strongest independent predictors of COVID-19 mortality. Patients aged 75 years and older have significantly higher risks of death compared to younger cohorts, partly due to immune senescence and a greater prevalence of comorbidities (2, 3).

Comorbid conditions such as chronic kidney disease, cardiovascular disease, diabetes mellitus, and hypertension also substantially increase the risk of in-hospital mortality. Studies across varied populations have confirmed the additive effect of multiple comorbidities on COVID-19 outcomes (4–6).

Laboratory markers measured at admission have shown high predictive value for in-hospital mortality. Blood urea nitrogen (BUN), C-reactive protein (CRP), lactate dehydrogenase (LDH), serum creatinine, and oxygen saturation levels are commonly elevated in non-survivors, signaling renal dysfunction, inflammation, and hypoxia, respectively (4, 7, 8).

Machine learning approaches have recently been employed to predict COVID-19 mortality with high accuracy, identifying variables such as oxygen saturation, renal markers, consciousness level, and comorbidity load as top predictors (7, 9).

Despite this growing body of global literature, evidence specific to the West Bank, Palestine remains sparse. The regional context, including different population health profiles and healthcare resource constraints, necessitates localized research. Therefore, this retrospective cohort study aims to identify key predictors of in-hospital mortality among COVID-19 patients in West Bank hospitals during the second wave of the pandemic. These findings will help inform clinical decision-making and policy planning in similarly resource-limited settings.

## Methods

### Study design

This study employed a retrospective cohort design, utilizing medical records of patients with confirmed COVID-19 who were admitted between November 1, 2020, and February 1, 2021. This period corresponded to the peak of the second COVID-19 wave in Palestine, during which hospitalization rates and mortality were at their highest, allowing us to examine predictors of in-hospital mortality under conditions of maximal healthcare system strain. The retrospective cohort approach was selected for its efficiency in terms of time and cost, allowing analysis of pre-existing clinical data without the need for prospective patient follow-up. Cohort designs also enable the evaluation of multiple exposures and outcomes concurrently (37).

### Study setting

The study was conducted across six governmental hospitals in the West Bank, Palestine, each designated as a COVID-19 treatment center with general wards and ICU facilities. These hospitals varied in capacity, ICU bed numbers, mechanical ventilation availability, and staffing ratios, reflecting the heterogeneous resource distribution across the Palestinian healthcare system.

### Sampling and sample size

A purposive sampling strategy was employed to select 200 patient records from the six hospitals, proportionally based on data availability. The minimum required sample size was 138 patients (calculated using a 1:1 group ratio, hazard ratio of 2, 80% power, and 5% type I error rate). Oversampling accounted for incomplete or missing data and improved statistical robustness.

### Study population

The target population consisted of adults (≥18 years) with laboratory-confirmed COVID-19 via PCR testing who were admitted to either general COVID-19 wards or intensive care units (ICUs) during the specified study period. All eligible patients were included as part of a single retrospective cohort, and information on ward type (general or ICU) was recorded for descriptive and analytical purposes.

### Inclusion and exclusion criteria

The inclusion criteria for this study were as follows: patients aged 18 years or older, with a confirmed COVID-19 diagnosis verified through PCR testing, who were admitted to either the intensive care unit (ICU) or general COVID-19 inpatient wards, and whose medical records were documented in the Palestinian Health Information System (HIS).

Conversely, patients were excluded if they were not admitted to inpatient care (for example, those discharged directly from the emergency department) or if their medical records were missing essential data such as demographic information, clinical presentation, laboratory test results, or treatment details.

### Variable definitions and measurements

- **Obesity** was defined as **BMI** **≥** **30 kg/m**^2^, calculated from height and weight documented on admission by nursing staff.
- **Hypertension** was defined as either:
  ° A documented prior diagnosis in the medical record, or
  ° Antihypertensive medication use prior to admission. Patients were further categorized into **controlled** (blood pressure < 140/90 mmHg at admission) and **uncontrolled** hypertension.
- **Immunocompromised status** included primary or secondary immunodeficiency, solid organ transplantation, active chemotherapy, long-term corticosteroid therapy (>10 mg/day prednisone equivalent for >14 days), or other immunosuppressive drugs.
- **Oxygen saturation (SpO**_**2**_**)**: Measured at admission using pulse oximetry on **room air** when possible. SpO_2_ < 94% was classified as abnormal. For patients already on oxygen therapy, the device and flow rate were recorded.
- **Partial pressure of oxygen (PaO**_**2**_**)**: Obtained from arterial blood gas analysis.
- **Creatinine levels** and other laboratory markers were obtained at **admission only**; subsequent in-hospital changes were not included in the primary analysis.
- **Oxygen therapy**: Type and intensity were recorded, including low-flow oxygen, high-flow nasal cannula (HFNC), continuous positive airway pressure (CPAP), and invasive mechanical ventilation, along with duration where available.
- **Chest CT scans**: The number performed, hospital location, and indications (e.g., suspected pulmonary embolism, severity assessment) were recorded. CT availability was not uniform across all sites.

### Data collection

Data were collected using a structured abstraction tool based on WHO COVID-19 case record forms, reviewed by two senior clinicians in governmental COVID-19 centers. Variables included demographic (age, sex, marital status, and hospital), clinical (BMI ≥ 30 kg/m^2^ for obesity, measured on admission; hypertension—prior diagnosis or current antihypertensive use, classified as controlled < 140/90 mmHg or uncontrolled; and immunocompromised status—primary/secondary immunodeficiency, transplant, chemotherapy, long-term corticosteroids ≥10 mg/day prednisone equivalent >14 days, or other immunosuppressants), and admission vital signs (temperature, respiratory rate, heart rate, blood pressure, oxygen saturation). Oxygen saturation was measured on room air when possible (< 94% abnormal); for patients on oxygen, delivery method and flow were recorded. PaO_2_ was taken from arterial blood gases when available. Laboratory markers (creatinine, random blood sugar, inflammatory markers) were from admission only. Radiology data included number and indication for chest CT scans; availability varied by hospital. Treatment data covered antimicrobials, anticoagulants, corticosteroids, and immunomodulators, noting drug type, dose, and duration where recorded. Hydrocortisone (50–100 mg IV q6–8h) and dexamethasone (6 mg daily) were documented separately. Oxygen therapy modalities (low-flow, high-flow nasal cannula, CPAP, invasive ventilation) and duration were recorded. Two trained researchers independently abstracted and cross-checked data, resolving discrepancies by consensus; unclear data were clarified via hospital records.

### Ethical considerations

The study protocol received ethical approval from the Institutional Review Board (IRB) of An-Najah National University, the Master of Public Health Program's scientific committee, and the Graduate Studies Research Council. All data were handled confidentially and used solely for research purposes. Participants or their legal guardians were informed of the study objectives, and consent was obtained where applicable.

### Statistical analysis

Data were analyzed using SPSS version 20. Descriptive statistics were used to summarize the population's demographic, clinical, and lab features. Associations between variables and patient outcomes were examined using Chi-square tests for categorical data, and *t-*tests or Mann-Whitney U tests for continuous data. Significant predictors from bivariate analysis were included in logistic regression to identify independent mortality predictors, with odds ratios (ORs) and 95% confidence intervals (CIs) reported. Additionally, Cox regression (univariate and multivariate) was used to assess factors affecting time to death, with hazard ratios (HRs) and 95% CIs provided.

## Results

### Descriptive analysis of study participants

A total of 200 confirmed COVID-19 patients admitted to six governmental hospitals in the West Bank, Palestine, were included in this retrospective cohort study. These patients were hospitalized between November 1, 2020, and February 1, 2021, during the height of the second wave of the pandemic. Of the total participants, 120 (60.0%) were female and 80 (40.0%) were male. The patients' ages ranged from 25 to over 65 years, with the largest age group being 35–44 years (27.5%), followed by the 45–54 and 55–64 age groups (both 19.5%). Of the total, 92 patients (46.0%) required ICU admission during hospitalization, while 108 (54.0%) were managed entirely in general wards. The in-hospital mortality rate among ICU-admitted patients was 68.5% compared to 21.3% among those treated in general wards, with a statistically significant association between ICU admission and mortality (χ^2^ = 43.22, *p* < 0.001).

The majority of the patients were married (51.5%), while 39.0% were single, and smaller proportions were divorced (4.0%) or widowed (5.5%). Regarding occupation, 36.5% were professional employees, 23.0% were craft workers, and 30.5% were unemployed, with only 10.0% working in the medical field. In terms of educational background, most patients had completed high school (34.5%) or held a diploma or bachelor's degree (30.0%), while 31.5% had primary education and 4.0% were illiterate.

### Demographic characteristics and in-hospital mortality

Table 1 presents the comparison between demographic variables and in-hospital mortality among 200 hospitalized COVID-19 patients in the West Bank. Several demographic factors were significantly associated with mortality outcomes.

**Table 1:** Comparison of demographic characteristics of COVID-19 patients participants based on outcome (discharge, death).

**Variable**	**Total *n* (%)**		**End point**		***χ^2^* (*p* value)**
		**Total**	**Discharge**	**Death**	
		***n*** **(%)**	***n*** **(%)**	***n*** **(%)**	
Hospital	Dura	44 (22.0)	17 (38.6)	27 (61.4)	**10.805 (0.055)**
	Military	20 (10.0)	11 (55.0)	9 (45.0)	
	Red crescent	11 (5.5)	9 (81.8)	2 (18.2)	
	Hugo Chavez	21 (10.5)	11 (52.4)	10 (47.6)	
	Tubas	41 (20.5)	26 (63.4)	15 (36.6)	
	Bethlehem	63 (31.5)	40 (63.5)	23 (36.5)	
Age (year)	25–34	30 (15.0)	16 (53.3)	14 (46.7)	0.508 (0.973)
	35–44	55 (27.5)	31 (56.4)	24 (43.6)	
	45–54	39 (19.5)	22 (56.4)	17 (43.6)	
	55–64	39 (19.5)	24 (61.5)	15 (38.5)	
	Above 65	37 (18.5)	21 (56.8)	16 (43.2)	
Gender	Male	80 (40.0)	47 (58.8)	33 (41.3)	0.167 (0.683)
	Female	120 (60.0)	67 (55.8)	53 (44.2)	
Marital status	Single	78 (39.0)	54 (69.2)	24 (30.8)	**10.893 (0.012)**
	Married	103 (51.5)	54 (52.4)	49 (47.6)	
	Divorced	8 (4.0)	2 (25.0)	6 (75.0)	
	Widowed	11 (5.5)	4 (36.4)	7 (63.6)	
Nature of work	Professional employee	73 (36.5)	49 (67.1)	24 (32.9)	**13.978 (0.003)**
	Graftsman	46 (23.0)	28 (60.9)	18 (39.1)	
	Medical community	20 (10.0)	14 (70.0)	6 (30.0)	
	Not work	61 (30.5)	23 (37.7)	38 (62.3)	
Monthly income (shekel)	Below 1,500	71 (35.5)	38 (53.5)	33 (46.5)	3.140 (0.208)
	1,500–3,000	67 (33.5)	44 (65.7)	23 (34.3)	
	3,000–4,500	62 (31.0)	32 (51.6)	30 (48.4)	
Education level	Illiterate	8 (4.0)	1 (12.5)	7 (87.5)	6.849 (0.077)
	Primary school	63 (31.5)	36 (57.1)	27 (42.9)	
	High school	69 (34.5)	41 (59.4)	28 (40.6)	
	Diploma \B.A	60 (30.0)	36 (60.0)	24 (40.0)	
Blood group	A+	32 (16.0)	12 (37.5)	20 (62.5)	**13.637 (0.058)**
	A^−^	20 (10.0)	13 (65.0)	7 (35.0)	
	AB+	17 (8.5)	7 (41.2)	10 (58.8)	
	AB^−^	21 (10.5)	9 (42.9)	12 (57.1)	
	O^−^	22 (11.0)	16 (72.7)	6 (27.3)	
	O+	39 (19.5)	24 (61.5)	15 (38.5)	
	B+	27 (13.5)	18 (66.7)	9 (33.3)	
	B^−^	22 (11.0)	15 (68.2)	7 (31.8)	
Place of residence	City	66 (33.0)	36 (54.5)	30 (45.5)	3.712 (0.156)
	Village	71 (35.5)	36 (50.7)	35 (49.3)	
	Refugee camps	63 (31.5)	42 (66.7)	21 (33.3)	
Smoking status	Smoking	62 (31.0)	37 (59.7)	25 (40.3)	4.526 (0.104)
	Ex-smokers	77 (38.5)	37 (48.1)	40 (51.9)	
	Non-smokers	61 (30.5)	40 (65.6)	21 (34.4)	
Obesity	Yes	64 (32.0)	20 (31.3)	44 (68.8)	**25.461 (0.000)**
	No	136 (68.0)	94 (69.1)	42 (30.9)	
Pregnancy	Yes	21 (17.5)	4 (19.0)	17 (81.0)	**13.968 (0.000)**
	No	99 (82.5)	63 (63.6)	36 (36.4)	
	**Mortality**	**N**	**Mean**	**Std. D**	**U (*****p*** **value)**
Weeks of pregnancy	Alive	4	30.0000	7.07107	19.00 (0.167)
	Died	17	34.4118	1.69775	

ICU, intensive care unit; U, Mann-Whitney U value. Bold values indicate statistically significant results (*p* < 0.05).

Marital status was found to be a statistically significant predictor of in-hospital death (χ^2^ = 10.893, *p* = 0.012). The mortality rate was highest among divorced (75.0%) and widowed patients (63.6%), compared to 47.6% among married and 30.8% among single individuals. These findings suggest that the absence of marital or family support may influence psychological resilience and access to timely healthcare, ultimately impacting survival.

Occupational status also showed a strong and statistically significant association with mortality (χ^2^ = 13.978, *p* = 0.003). Patients who were not employed exhibited a 62.3% mortality rate, considerably higher than professional employees (32.9%), craft workers (39.1%), and individuals working in the medical field (30.0%). This indicates that socioeconomic disparities, possibly linked to underlying health conditions and healthcare access, could be influencing mortality risk.

Obesity emerged as one of the strongest predictors of in-hospital death (χ^2^ = 25.461, *p* < 0.001). Among obese patients, 68.8% died, compared to just 30.9% among those with normal weight. This substantial difference reinforces global evidence on the adverse impact of obesity on COVID-19 outcomes, likely due to inflammation, impaired lung function, and metabolic comorbidities.

Pregnancy was also significantly associated with higher mortality (χ^2^ = 13.968, *p* < 0.001). Pregnant women had an 81.0% mortality rate, compared to 36.4% among non-pregnant women. These results highlight the clinical vulnerability of pregnant women with COVID-19 and the need for prioritized care in this subgroup.

Although blood group was not statistically significant overall (χ^2^ = 13.637, *p* = 0.058), a notable variation was observed among subgroups. Patients with blood group A^+^ had the highest mortality rate (62.5%), while those with O^−^ and B^−^ had the lowest rates at 27.3 and 31.8%, respectively. This trend aligns with some international studies suggesting a possible link between ABO blood type and COVID-19 severity.

Other variables, such as age, gender, monthly income, education level, residence location, and smoking status, did not show statistically significant associations with mortality in this cohort (*p* > 0.05; see Table 1).

These findings suggest that specific demographic characteristics—particularly marital status, employment, obesity, pregnancy, and blood group—are important predictors of in-hospital mortality among COVID-19 patients and should be considered in clinical risk stratification and public health planning.

### Medical history and in-hospital mortality

Table 2 presents a comparative analysis between the medical history of COVID-19 patients and their in-hospital mortality outcomes. This section aimed to explore whether pre-existing chronic diseases contributed to increased mortality risk among hospitalized patients. Contrary to trends reported in broader literature, the findings from this study indicate that no statistically significant associations were observed between any of the assessed comorbidities and the risk of in-hospital death.

**Table 2:** Comparison of medical history of COVID-19 patients participants based on outcome (Discharge, Death).

**Medical history**	**Status (Yes/No)**	**Total *n* (%)**	**End point**		**χ2 (*p* value)**
			**Discharge**	**Death**	
Cardiac disease	Yes	48 (24.0)	29 (60.4)	19 (39.6)	0.301 (0.583)
	No	152 (76.0)	85 (65.9)	67 (44.1)	
Chronic lung disease	Yes	15 (7.5)	8 (53.3)	7 (46.7)	0.089 (0.766)
	No	185 (92.5)	106 (57.3)	79 (42.7)	
Diabetes	Yes	70 (35.0)	41 (58.6)	29 (41.4)	0.109 (0.742)
	No	130 (65.0%)	73 (56.2)	57 (43.8)	
Hypertension	Yes	62 (31.0%)	35 (56.5)	27 (43.5)	0.011 (0.916)
	No	138 (69.0%)	79 (57.2)	59 (42.8)	
Chronic liver	Yes	5 (2.5%)	2 (40.0)	3 (60.0)	0.605 (0.437)
	No	195 (97.5%)	112 (57.4)	83 (42.6)	
Chronic renal	Yes	3 (1.5%)	2 (66.7)	1 (33.3)	0.116 (0.733)
	No	197 (98.5%)	112 (56.9)	85 (43.1)	
Immunosuppressed	Yes	7 (3.5%)	5 (71.4)	2 (28.6)	0.616 (0.432)
	No	193 (96.5%)	109 (56.5)	84 (43.5)	
On dialysis	Yes	3 (1.5%)	2 (66.7)	1 (33.3)	0.116 (0.733)
	No	197 (98.5%)	112 (56.9)	85 (43.1)	
Blood diseases	Yes	10 (5.0%)	4 (40.0)	6 (60.0)	1.241 (0.265)
	No	190 (95.0%)	110 (57.9)	80 (42.1)	
Nervous diseases	Yes	3 (1.5%)	2 (66.7)	1 (33.3)	0.116 (0.733)
	No	197 (98.5%)	112 (56.9)	85 (43.1)	
Endocrine diseases	Yes	3 (1.5%)	1 (33.3)	2 (66.7)	0.696 (0.404)
	No	197 (98.5%)	113 (57.4)	84 (42.6)	

ICU, intensive care unit, χ^2^, Chi-square test statistic. Bold values indicate statistically significant results (*p* < 0.05).

Among patients with cardiovascular disease, 39.6% died during hospitalization, while 44.1% of those without such a history also died (χ^2^ = 0.301, *p* = 0.583). Similarly, patients with chronic lung diseases had a mortality rate of 46.7%, compared to 42.7% among those without pulmonary conditions (χ^2^ = 0.089, *p* = 0.766). These findings suggest that cardiac and respiratory history alone did not meaningfully impact mortality risk in this cohort.

Regarding diabetes mellitus, 41.4% of diabetic patients died, which was slightly lower than the 43.8% mortality observed among non-diabetics (χ^2^ = 0.109, *p* = 0.742). A similar trend was observed for hypertension, where the mortality rates were nearly identical between hypertensive (43.5%) and non-hypertensive (42.8%) individuals (χ^2^ = 0.011, *p* = 0.916). These results imply that metabolic comorbidities did not significantly influence survival in the hospitalized population studied.

Other pre-existing conditions—including chronic liver disease (60.0% mortality), chronic kidney disease, immunosuppression, blood disorders, nervous system diseases, and endocrine disorders—also did not show statistically significant relationships with death. All of these conditions had very small sample sizes (ranging from 1.5% to 5.0% of the cohort), which may have limited the statistical power to detect true differences.

In summary, although many patients had one or more underlying health conditions, the presence of specific chronic diseases such as cardiovascular, pulmonary, metabolic, renal, or hepatic conditions did not significantly predict in-hospital mortality among COVID-19 patients in this sample. This suggests that other factors—such as acute clinical status, demographic variables like obesity and pregnancy, and biochemical markers—may have had a more decisive influence on patient outcomes.

### Clinical presentations and in-hospital mortality

The distribution of chief complaints among COVID-19 patients and their association with in-hospital mortality. This analysis aimed to determine whether presenting symptoms at admission could be used as predictors for patient outcomes.

Overall, the analysis revealed that none of the individual clinical symptoms showed a statistically significant association with in-hospital mortality. However, some trends were observed that merit consideration.

The most commonly reported symptoms among all participants included fever (85.0%), joint pain (80.0%), muscle pain (77.5%), cough (70.0%), and loss of taste or smell (70.0%). Mortality rates for these symptoms ranged from 14.0% (fever) to 31.4% (cough), but the differences between those who died and those discharged were not statistically significant. For instance, patients presenting with fever had a 14.0% mortality rate compared to 86.0% among those who did not report fever (χ^2^ = 0.130, *p* = 0.719).

Symptoms such as shortness of breath, diarrhea, vomiting, and conjunctivitis—frequently associated with more severe disease—were also not significantly linked to mortality in this sample. For example, vomiting was reported by 30.0% of patients and showed a non-significant trend toward higher mortality (58.1% vs. 33.7% in those without vomiting; χ^2^ = 4.763, *p* = 0.092). Similarly, shortness of breath was reported by 40.0% of patients and was slightly more common in non-survivors (57.0%) than survivors (43.0%), but without statistical significance (χ^2^ = 0.575, *p* = 0.448).

Additional non-significant findings included complaints of abdominal pain, malaise, nausea, and fatigue, each showing close mortality percentages across groups with or without the symptom.

Radiological findings also offered limited predictive value. On chest X-ray, bilateral patchy shadowing was observed in 47.4% of patients, and lung infiltration in 52.6%, but these patterns were not statistically different between outcome groups (χ^2^ = 0.418, *p* = 0.518). However, CT scan findings showed a statistically significant difference (χ^2^ = 9.000, *p* = 0.029), suggesting some radiologic patterns may correlate with mortality risk. Specifically, patients with certain abnormal CT endpoints showed worse outcomes, although this was based on a small subgroup.

In terms of laboratory markers, C-reactive protein (CRP) was positive in 100% of cases and therefore offered no discriminatory value. Troponin I, a marker of cardiac injury, was elevated in 82.0% of patients, but the difference between survivors (77.9%) and non-survivors (85.1%) was not statistically significant (χ^2^ = 1.712, *p* = 0.191). In conclusion, although several symptoms and imaging features were common among patients, none of the chief complaints individually predicted in-hospital mortality, with the exception of a significant CT scan pattern association, warranting further exploration. These results emphasize the importance of integrating symptoms with laboratory and radiological data to improve risk stratification.

### Hemodynamic and laboratory findings in relation to in-hospital mortality

Table 4 presents a comparison of hemodynamic parameters and laboratory test results between COVID-19 patients who survived and those who died during hospitalization. The aim was to identify early clinical or biochemical indicators associated with poor outcomes.

Among all measured variables, three were found to have statistically significant associations with mortality: partial pressure of oxygen (PaO_2_), serum creatinine (CRE), and random blood sugar (RBS).

Partial pressure of oxygen (PaO_2_) was significantly lower in non-survivors compared to survivors (mean 97.45 mmHg vs. 103.29 mmHg, *p* = 0.044), suggesting that impaired oxygenation upon admission was associated with a higher risk of death.

Serum creatinine levels were also significantly lower among deceased patients (mean 1.26 mg/dL) than those who survived (mean 1.47 mg/dL), with a *p* value of 0.015. This may indicate that patients who died had lower muscle mass or were in a different metabolic state, although the clinical significance of this finding requires further investigation.

Random blood sugar (RBS) showed a highly significant difference between the two groups. Survivors had significantly higher mean RBS levels (207.96 mg/dL) compared to non-survivors (173.50 mg/dL, *p* = 0.001). While counterintuitive, this may reflect a stress hyperglycemia response in survivors or different treatment responses.

Other parameters showed notable trends but did not reach statistical significance. For example, temperature was marginally higher in non-survivors (*p* = 0.052), and blood urea nitrogen (BUN) and prothrombin time (PT) also approached significance with *p* values of 0.063 and 0.065, respectively. Both of these may warrant attention in larger studies.

No statistically significant differences were observed in common inflammatory markers such as C-reactive protein (CRP), D-dimer, ferritin, liver enzymes (ALT, AST), or white blood cell counts, although trends in elevated WBC and neutrophils in non-survivors were noted (mean WBC: 14.93 vs. 14.09, *p* = 0.088; neutrophils: 8.15 vs. 6.84, *p* = 0.140).

In conclusion, Table 3 highlights that lower PaO_2_, lower serum creatinine, and lower RBS levels were statistically associated with increased mortality risk in hospitalized COVID-19 patients. These parameters may serve as early indicators of severe disease progression and require close monitoring in clinical practice.

**Table 3:** Comparison of hemodynamics and lab result between alive and died COVID-19 patients.

**Demographic variable**	**Mortality**	***N***	**Mean**	**Std. D**	***T***	***P* value**
Highest temperature	Alive	114	38.54	0.95	0.467	0.641
	Died	86	38.48	0.80		
O2 saturation	Alive	114	92.39	3.31	−1.626	0.106
	Died	86	93.17	3.46		
Temperature	Alive	114	37.95	0.81	−1.955	**0.052**
	Died	86	38.17	0.77		
Heart Rate	Alive	114	93.41	17.62	1.067	0.287
	Died	86	90.80	16.73		
Systolic BP	Alive	114	123.61	19.56	−1.330	0.185
	Died	86	127.47	20.87		
Diastolic BP	Alive	114	68.92	14.65	−1.544	0.124
	Died	86	72.35	16.19		
Resp. Rate	Alive	114	21.75	4.31	0.780	0.437
	Died	86	21.28	4.10		
PaO_2_	Alive	114	103.29	20.47	2.026	**0.044**
	Died	86	97.45	20.00		
PaCO_2_	Alive	114	40.52	5.20	−0.263	0.793
	Died	86	40.72	5.37		
PH	Alive	114	7.39	0.10	0.473	0.637
	Died	86	7.38	0.09		
HCO_3_	Alive	114	24.05	2.03	−0.328	0.744
	Died	86	24.16	2.82		
ALT	Alive	114	45.48	19.47	1.354	0.177
	Died	86	41.76	19.09		
AST	Alive	114	38.13	16.71	−0.226	0.821
	Died	86	38.63	14.29		
BUN	Alive	114	31.45	18.00	1.873	**0.063**
	Died	86	26.75	17.24		
CRE	Alive	114	1.47	0.58	2.448	**0.015**
	Died	86	1.26	0.59		
D dimer	Alive	114	563.97	189.07	−1.033	0.305
	Died	86	770.26	1,844.82		
PTT	Alive	114	40.36	15.77	−0.333	0.739
	Died	86	41.16	17.76		
PT	Alive	114	28.90	10.50	1.854	**0.065**
	Died	86	26.07	10.84		
INR	Alive	114	2.08	0.81	0.980	0.328
	Died	86	1.95	0.94		
Hb	Alive	114	13.17	1.75	1.003	0.317
	Died	86	12.88	2.23		
Lymphocyte	Alive	114	1.41	0.55	−0.878	0.382
	Died	86	1.66	2.66		
WBC	Alive	114	14.09	3.41	−1.715	0.088
	Died	86	14.93	3.46		
Platelet	Alive	114	314.52	84.62	0.151	0.880
	Died	86	312.71	83.03		
Neutrophil	Alive	114	6.84	2.91	−1.488	0.140
	Died	86	8.15	7.80		
HbA1c	Alive	114	1.75	0.43	−0.810	0.419
	Died	86	1.80	0.40		
RBS	Alive	114	207.96	87.74	3.269	**0.001**
	Died	86	173.50	61.21		
LDH	Alive	114	270.33	71.95	−1.280	0.202
	Died	86	284.38	87.86		
Ferritin	Alive	114	660.51	204.47	0.467	0.641
	Died	86	632.51	248.56		

PaO_2_, Partial pressure of oxygen; PaCO_2_, Partial pressure of carbon dioxide; BP, Blood pressure; HR, Heart rate; Resp. Rate, Respiratory rate; ALT, Alanine aminotransferase; AST, Aspartate aminotransferase; BUN, Blood urea nitrogen; CRE, Creatinine; PT, Prothrombin time; PTT, Partial thromboplastin time; INR, International normalized ratio; Hb, Hemoglobin; WBC, White blood cell count; RBS, Random blood sugar; HbA1c, Hemoglobin A1c; LDH, Lactate dehydrogenase; CRP, C-reactive protein; HCO3, Bicarbonate; t, Independent samples t-tests. Bold values indicate statistically significant results (*p* < 0.05).

### Treatment and medication use in relation to in-hospital mortality

Table 4 compares the types of medications administered to hospitalized COVID-19 patients and their corresponding outcomes, specifically survival vs. death. Among the drugs evaluated, three showed statistically significant associations with mortality: vancomycin, ceftriaxone, and Actemra (tocilizumab).

**Table 4:** Comparison of drugs used by COVID-19 patients' participants based on outcome (Discharge, Death).

**Clinical characteristic**		**Mortality**		***X*^2^ (*p* value)**
		**Alive**	**Died**	
Vancomycin	No	77 (64.2%)	43 (35.8%)	**6.287 (0.012)**
	Yes	37 (46.3%)	43 (53.8%)	
Meropenem	No	75 (61.0%)	48 (39.0%)	2.060 (0.151)
	Yes	39 (50.6%)	38 (49.4%)	
Ciprofloxacin	No	113 (57.4%)	84 (42.6%)	0.696 (0.404)
	Yes	1 (33.3%)	2 (66.7%)	
Ceftriaxone	No	71 (63.4%)	41 (36.6%)	4.244 (0.039)
	Yes	43 (48.9%)	45 (51.1%)	
Tazocin	No	101 (58.0%)	73 (42.0%)	0.327 (0.568)
	Yes	13 (52.0%)	12 (48.0%)	
Levofloxacin	No	17 (54.8%)	14 (45.2%)	0.070 (0.791)
	Yes	97 (57.4%)	72 (42.6%)	
Azithromycin	No	5 (35.7%)	964.3% ()	2.783 (0.095)
	Yes	109 (58.6%)	77 (41.4%)	
Hydrocortisone	No	51 (59.3%)	35 (40.7%)	0.326 (0.568)
	Yes	63 (55.3%)	51 (44.7%)	
Cough syrup	No	33 (56.9%)	25 (43.1%)	0.000 (0.985)
	Yes	81 (57.0%)	61 (43.0%)	
Vitamin C	No	35 (57.4%)	26 (42.6%)	0.005 (0.943)
	Yes	79 (56.8%)	60 (43.2%)	
Vitamin D	No	24 (63.2%)	14 (36.8%)	0.726 (0.394)
	Yes	90 (55.6%)	72 (44.4%)	
Zinc	No	20 (50.0%)	20 (50.0%)	1.000 (0.317)
	Yes	94 (58.8%)	66 (41.3%)	
Acetylcysteine	No	80 (54.1%)	68 (45.9%)	2.016 (0.156)
	Yes	34 (65.4%)	18 (34.6%)	
Enoxaparin	No	5 (38.5%)	8 (61.5%)	1.950 (0.163)
	Yes	109 (58.3%)	78 (41.7%)	
PI	No	3 (75.0%)	1 (25.0%)	0.540 (0.463)
	Yes	111 (56.6%)	85 (43.4%)	
Corticosterids Neb	No	5 (71.4%)	2 (28.6%)	0.616 (0.432)
	Yes	109 (56.5%)	84 (43.5%)	
Paracetamol	No	32 (51.6%)	30 (48.4%)	1.064 (0.302)
	Yes	82 (59.4%)	56 (40.6%)	
Colchicine	No	102 (56.0%)	80 (44.0%)	0.754 (0.385)
	Yes	12 (66.7%)	6 (33.3%)	
Actemra	No	101 (54.0%)	86 (46.0%)	**10.489 (0.001)**
	Yes	13 (100.0%)	0 (0.0%)	

PI, Proton Inhibitors; Neb, Nebulizer; IV, Intravenous; Actemra, Tocilizumab; X^2^ (p value), Chi-square test statistic and associated p-value; CRP, C-reactive protein; ICU, Intensive Care Unit. Bold values indicate statistically significant results (*p* < 0.05).

Vancomycin use was significantly associated with higher mortality. Patients who received vancomycin had a mortality rate of 53.8% compared to 35.8% in those who did not receive it (*p* = 0.012). This may reflect its use in more severe cases or those with suspected bacterial co-infection, rather than a direct harmful effect.

Similarly, ceftriaxone use was also linked to increased mortality. Among those who received it, 51.1% died, vs. 36.6% of those who did not receive the drug (*p* = 0.039). Like vancomycin, this association may be indicative of its use in more critically ill patients. In unadjusted analysis, antibiotic use appeared associated with higher mortality (OR = 2.10, 95% CI: 1.15–3.82, *p* = 0.016). However, after adjusting for ICU admission status, this association was no longer statistically significant (AOR = 1.28, 95% CI: 0.65–2.50, *p* = 0.48), suggesting that the initial finding was likely confounded by disease severity.

On the other hand, Actemra (tocilizumab) showed a protective association. All 13 patients who received Actemra survived, with 0% mortality compared to a 46% mortality rate among those who did not receive the medication (*p* = 0.001). This finding supports the potential role of Actemra in reducing the risk of death among hospitalized COVID-19 patients, likely due to its immunomodulatory effect on cytokine storm.

Other drugs, including meropenem, ciprofloxacin, tazocin, levofloxacin, azithromycin, hydrocortisone, colchicine, vitamins (C and D), zinc, and anticoagulants like enoxaparin, did not show statistically significant differences in mortality. While some showed trends—such as higher survival in patients receiving acetylcysteine or vitamin D—these did not reach statistical significance.

In summary, Table 4 demonstrates that the use of vancomycin and ceftriaxone was associated with higher in-hospital mortality, likely due to disease severity, while Actemra was strongly associated with survival. These findings may guide clinicians in risk stratification and therapeutic decision-making during COVID-19 management.

### Multivariate logistic regression analysis of predictors of in-hospital mortality among COVID-19 patients

The logistic regression analysis in Table 6 identified several predictors significantly associated with in-hospital mortality among COVID-19 patients. The analysis included variables found significant in bivariate analysis and used the forward stepwise method.

Hospital type was a significant predictor of mortality (*p* = 0.049). Patients admitted to Dura hospital were over three times more likely to die compared to patients admitted to other hospitals (OR = 3.257, 95% CI: 1.239–8.562). This may suggest differences in case severity, resource availability, or treatment protocols.

Marital status was also statistically significant overall (*p* = 0.028). However, within-group comparisons showed no specific marital category had a statistically significant odds ratio at the 0.05 level. For instance, divorced patients had an OR = 3.012 (95% CI: 0.266–34.167), and single patients had a lower odds of death (OR = 0.239, 95% CI: 0.047–1.229), although both results were not statistically significant individually (*p* > 0.05).

Obesity was a strong and statistically significant predictor of death (*p* < 0.001). Obese patients were more than five times more likely to die compared to non-obese patients (OR = 5.731, 95% CI: 2.386–13.763), highlighting the well-documented role of obesity as a severe risk factor in COVID-19 outcomes.

Other variables, including nature of work, smoking status, blood group, and pregnancy, were not found to be statistically significant predictors of mortality in the final model (p > 0.05). Notably, pregnancy showed an increased odds ratio of 2.046, but this was not statistically significant (*p* = 0.347, 95% CI: 0.461–9.079), likely due to the limited sample size of pregnant women.

These findings suggest that hospital of admission and obesity were the most consistent and statistically significant predictors of in-hospital mortality among COVID-19 patients in this cohort. The adjusted hazard ratios for predictors of in-hospital mortality are summarized in Table 5.

**Table 5:** Predictors of Death among COVID-19 patients.

**Predictor variable**	**B**	**S.E**.	**Wald**	***df***	**Sig**.	**Exp (B)**	**95% CI for Exp (B)**	
							**Lower**	**Upper**
Hospital			11.104	5	**0.049**			
*Dura*	1.181	0.493	5.733	1	**0.017**	3.257	1.239	8.562
*Military*	−0.726	0.677	1.149	1	0.284	0.484	0.128	1.824
*Red crescent*	−1.126	0.934	1.452	1	0.228	0.324	0.052	2.025
*Hugo Chavez*	−0.193	0.641	0.091	1	0.763	0.825	0.235	2.894
*Tubas*	−0.044	0.511	0.007	1	0.932	0.957	0.352	2.606
Marital status			9.136	3	**0.028**			
*Single*	−1.430	0.835	2.935	1	0.087	0.239	0.047	1.229
*Married*	−0.855	0.835	1.048	1	0.306	0.425	0.083	2.185
*Divorced*	1.103	1.239	0.792	1	0.374	3.012	0.266	34.167
Nature of work	−0.190	0.202	0.889	1	0.346	0.827	0.557	1.228
Blood group			11.498	7	0.118			
Smoking status	−0.135	0.229	0.346	1	0.556	0.874	0.558	1.369
Obesity	1.746	0.447	15.255	1	**0.000**	5.731	2.386	13.763
Pregnancy	0.716	0.760	0.886	1	0.347	2.046	0.461	9.079
Constant	−0.746	1.166	0.409	1	0.523	0.474		

B, Regression coefficient; S.E., Standard Error; Wald, Wald test statistic; df, Degrees of Freedom; Sig., Significance level (p-value); Exp (B), Exponentiated coefficient, representing the odds ratio; 95% CI for Exp (B), 95% Confidence Interval for the odds ratio. Bold values indicate statistically significant results (*p* < 0.05).

### Univariable cox regression analysis of factors associated with COVID-19 mortality

The univariate Cox regression analysis presented in Table 6 identified several clinical and demographic factors significantly associated with mortality among hospitalized COVID-19 patients.

**Table 6:** Univariate cox-regression analysis of factors contributing to mortality.

**Laboratory parameter**	**B**	**Wald**	***df***	**Sig**.	**Exp (B)**	**95.0% CI for Exp (B)**
Marital status		5.550	3	0.136		
*Single*	Ref					
*Married*	0.224	0.776	1	0.378	1.251	0.760– 2.060
*Divorced*	0.194	0.168	1	0.682	1.214	0.480–3.068
*Widowed*	1.020	5.543	1	**0.019**	2.773	1.186–6.481
Blood group		13.126	7	0.069		
*A+*	Ref					
*A^−^*	−0.428	0.919	1	0.338	0.652	0.272–1.563
*AB+*	−0.042	0.011	1	0.916	0.959	0.442–2.083
*AB^−^*	−0.340	0.835	1	0.361	0.712	0.343–1.476
*O^−^*	−1.262	6.346	1	**0.012**	0.283	0.106–0.756
*O+*	−0.400	1.318	1	0.251	0.670	0.338–1.327
*B+*	−1.016	6.174	1	**0.013**	0.362	0.163–0.807
*B^−^*	−0.891	3.977	1	**0.046**	0.410	0.171–0.985
Obesity	1.058	22.868	1	**0.000**	2.879	1.867–4.441
Pregnancy	0.802	8.338	1	**0.004**	2.231	1.294–3.845
Weeks of pregnancy	0.060	0.387	1	0.534	1.062	0.879–1.283
PaO_2_	−0.005	0.897	1	0.344	0.995	0.984–1.006
CRE	−0.391	3.430	1	0.064	0.677	0.448–1.023
RBS	−0.004	6.131	1	**0.013**	0.996	0.992–0.999
Temperature	0.321	5.581	1	**0.018**	1.378	1.056–1.798

B, Regression coefficient; Wald, Wald test statistic; df, Degrees of Freedom; Sig., Significance level (p-value); Exp (B), Exponentiated coefficient representing the hazard ratio (HR); CI, Confidence Interval; PaO_2_, Partial Pressure of Oxygen; CRE, Creatinine; RBS, Random Blood Sugar. Bold values indicate statistically significant results (*p* < 0.05).

Marital status had a borderline overall significance (*p* = 0.136). Notably, widowed patients were significantly more likely to die compared to single patients (HR = 2.773, 95% CI: 1.186–6.481, *p* = 0.019), indicating a potential impact of social support on survival outcomes.

Blood group was also close to overall significance (*p* = 0.069), with specific groups showing statistically significant associations. Patients with blood group O^−^ had a significantly lower risk of death (HR = 0.283, 95% CI: 0.106–0.756, *p* = 0.012), as did those with blood groups B^+^ (HR = 0.362, 95% CI: 0.163–0.807, *p* = 0.013) and B^−^ (HR = 0.410, 95% CI: 0.171–0.985, *p* = 0.046), when compared to blood group A^+^ (reference group).

Obesity emerged as a strong predictor of mortality (HR = 2.879, 95% CI: 1.867–4.441, *p* < 0.001), confirming its significant impact on disease outcome.

Pregnancy was another significant risk factor, with pregnant patients being over twice as likely to die compared to non-pregnant patients (HR = 2.231, 95% CI: 1.294–3.845, *p* = 0.004). However, gestational age (weeks of pregnancy) was not significantly associated with mortality (*p* = 0.534).

Several physiological and laboratory parameters were also assessed. Higher body temperature was significantly associated with increased mortality (HR = 1.378, 95% CI: 1.056–1.798, *p* = 0.018), while lower random blood sugar (RBS) levels were weakly associated with greater survival (HR = 0.996, 95% CI: 0.992–0.999, *p* = 0.013). Serum creatinine (CRE) also approached statistical significance, with higher levels tending toward a lower risk of death (HR = 0.677, 95% CI: 0.448–1.023, *p* = 0.064), though this did not reach conventional significance.

In summary, univariate analysis highlights widowed marital status, certain blood groups, obesity, pregnancy, elevated body temperature, and RBS as significant or near-significant predictors of mortality among COVID-19 patients.

### Multivariate cox proportional hazards model identifying predictors of time to in-hospital mortality among COVID-19 patients

Table 7 presents the findings from the multivariate Cox regression analysis aimed at identifying the most significant predictors of time to mortality among hospitalized COVID-19 patients. This model included variables found to be significant or near-significant in univariate analyses and controlled for multiple confounders simultaneously.

**Table 7:** Multivariate cox regression analysis of factors contributing to time in ward till mortality.

**Comorbidity**	**B**	**Wald**	**Sig**.	**Exp (B)**	**95.0% CI for Exp (B)**	
					**Lower**	**Upper**
Marital status	0.174	1.435	0.231	1.190	0.895	1.581
Blood group	−0.079	2.773	0.096	0.924	0.842	1.014
Obesity	0.980	14.670	**0.000**	2.664	1.614	4.399
Pregnancy	0.026	0.007	0.934	1.026	0.556	1.894
Temperature	0.239	3.231	0.072	1.270	0.979	1.647
RBS	−0.003	2.977	0.084	0.997	0.993	1.000

HR, Hazard Ratio; CI, Confidence Interval; RBS, Random Blood Sugar; Sig., Significance level (*p*-value); Exp (B), Exponentiated Coefficient, representing the hazard ratio; B, Unstandardized regression coefficient in Cox regression; Wald, Wald test statistic used to determine the significance of individual predictors. Bold values indicate statistically significant results (*p* < 0.05).

Obesity remained a strong and statistically significant independent predictor of earlier mortality (p < 0.001). Obese patients were approximately 2.66 times more likely to die earlier compared to non-obese patients (HR = 2.664, 95% CI: 1.614–4.399).

Other variables including marital status, blood group, pregnancy, temperature, and random blood sugar (RBS) did not reach statistical significance in the multivariate model. Marital status (*p* = 0.231), pregnancy (*p* = 0.934), and blood group (*p* = 0.096) all showed non-significant associations. However, temperature (HR = 1.270, *p* = 0.072) and RBS (HR = 0.997, *p* = 0.084) approached statistical significance and may warrant further investigation in larger cohorts.

These findings reaffirm the impact of obesity on COVID-19 mortality risk, even after adjusting for key demographic and clinical variables.

## Discussion

This retrospective cohort study aimed to identify demographic, clinical, laboratory, and treatment-related predictors of in-hospital mortality among COVID-19 patients admitted to six governmental hospitals in the West Bank, Palestine. The findings revealed several critical risk factors, including obesity, pregnancy, hospital type, marital status, and elevated temperature, as significant predictors of mortality. These observations are consistent with global literature and provide locally relevant insights for risk stratification, clinical decision-making, and targeted intervention in COVID-19 hospital management.

One of the most noteworthy findings was the significant association between marital status and in-hospital mortality. Divorced and widowed individuals demonstrated disproportionately high mortality rates compared to their married and single counterparts. This may reflect the psychosocial vulnerability and reduced support systems in these groups, which can delay healthcare seeking, hinder adherence to treatment, and exacerbate stress-related immunosuppression. Social isolation and mental health challenges during hospitalization have previously been associated with worse outcomes in pandemic settings (10–12). Healthcare providers are thus encouraged to consider psychosocial assessments as part of comprehensive COVID-19 care.

Although age and gender were not significant predictors in our multivariate analysis, this does not negate their established roles as risk factors. Numerous studies have demonstrated that advanced age and male gender are independently associated with higher COVID-19 mortality (13–15). The lack of statistical significance in our study may be attributable to the relatively younger age distribution of our cohort and an almost balanced gender representation. Nonetheless, age- and sex-specific interventions should still be considered in clinical practice.

Obesity emerged as a consistent and robust predictor of mortality across logistic and Cox regression models. Obese patients were more than five times as likely to die in-hospital and nearly three times as likely to experience earlier death compared to their non-obese counterparts. This is in line with a vast body of evidence showing that obesity impairs respiratory function, enhances systemic inflammation, and is often accompanied by comorbid conditions such as diabetes and hypertension that worsen COVID-19 outcomes (16–18). Moreover, obesity has been linked to mechanical limitations in ventilation, including decreased lung compliance, increased airway resistance, and impaired diaphragmatic excursion. Severe obesity is also associated with obesity hypoventilation syndrome and obstructive sleep apnea, both of which compound respiratory compromise in COVID-19 patients (19, 20).

Pregnancy was also observed to be associated with a notably higher risk of in-hospital mortality. In the univariate Cox regression analysis, pregnant patients had a mortality rate of 81%, substantially exceeding that of non-pregnant patients. However, this association did not remain statistically significant in multivariable models, likely due to the small number of pregnant participants. The observed pattern is consistent with prior studies suggesting that physiological and immunological changes during pregnancy—such as reduced functional lung capacity from uterine enlargement, increased oxygen demand, and a prothrombotic state—may increase susceptibility to severe outcomes in viral infections (21, 22). Factors such as reduced functional lung capacity due to uterine enlargement, increased oxygen demand, and prothrombotic state may contribute to adverse outcomes.

Although blood group was not a statistically significant predictor in multivariate models, univariate analyses revealed noteworthy trends. Patients with blood group A+ had the highest mortality rate, whereas those with O- had the lowest. These findings support hypotheses regarding immunologic interactions between ABO blood antigens and SARS-CoV-2 binding mechanisms, as proposed in earlier studies (23, 24).

Hospital type also significantly influenced mortality outcomes. Patients admitted to Dura Hospital were more than three times as likely to die compared to those admitted to other hospitals. This disparity may reflect variation in ICU bed availability, staff-to-patient ratios, hospital protocols, and surge capacity. As noted in previous global studies, healthcare infrastructure significantly impacts COVID-19 survival, and efforts to standardize hospital care across facilities are imperative (25, 26).

The study identified several clinical and laboratory indicators that were significantly associated with increased mortality. Among these, hypoxia (as measured by low PaO_2_), low serum creatinine levels, and abnormal RBS levels stood out. Hypoxemia is a well-documented marker of severe disease and is associated with increased need for ventilatory support and ICU admission (27). Lower serum creatinine may indicate reduced muscle mass due to chronic illness or malnutrition, while high levels suggest renal dysfunction—both conditions associated with higher mortality risk (28, 29). Unfortunately, data on Glomerular Filtration Rate (GFR) were not consistently available in our medical records, limiting our ability to incorporate this more sensitive marker of renal function into the analysis. Future studies should include GFR to better elucidate the relationship between kidney function and COVID-19 mortality.

Interestingly, lower RBS values were associated with higher mortality, which contradicts typical expectations. Some recent studies have shown that mild hyperglycemia, particularly in non-diabetic patients, may reflect an adaptive response to acute stress and improved metabolic reserve. Hypoglycemia, by contrast, can impair cognitive and physiological functioning, increasing the risk of poor outcomes (30, 31).

Temperature, another key finding, was marginally significant in both logistic and Cox models. Elevated temperature has been associated with cytokine dysregulation and can act as a proxy for severe systemic inflammation, which may drive adverse outcomes (32, 33).

The use of vancomycin and ceftriaxone was associated with increased mortality in our cohort. While this likely reflects confounding by indication—these antibiotics are often reserved for severe cases—it underscores the need for judicious antimicrobial stewardship and early intervention to prevent secondary bacterial infections. In our unadjusted analysis, antibiotic use appeared associated with higher mortality (OR = 2.10, 95% CI: 1.15–3.82, *p* = 0.016). However, after adjusting for ICU admission status as a proxy for disease severity, this association was no longer statistically significant (AOR = 1.28, 95% CI: 0.65–2.50, *p* = 0.48), supporting the conclusion that the initial association was likely driven by severity-related confounding rather than a direct harmful effect.

Conversely, patients treated with Actemra (tocilizumab), an interleukin-6 (IL-6) inhibitor, had significantly improved survival. This aligns with international findings that tocilizumab mitigates the cytokine release syndrome associated with critical COVID-19 cases, reducing inflammation and improving oxygenation (34–36).

A major strength of this study is its comprehensive analysis using multivariable models to identify independent predictors of mortality. The inclusion of a diverse patient population from six hospitals improves the generalizability of findings within the West Bank.

This study has several limitations that should be considered when interpreting the findings. First, the purposive, non-random sampling of patients may limit the generalizability of results to all hospitalized COVID-19 patients in Palestine. Second, small sample sizes in certain subgroups—particularly pregnant women—reduced statistical power to detect associations. Third, the retrospective design and reliance on medical records introduced the potential for misclassification or incomplete data, despite our efforts to cross-check and verify entries. Fourth, residual confounding is possible, especially for treatment-related variables, as certain medications (e.g., antibiotics) were more likely to be prescribed to patients with severe illness. Fifth, hospital-level differences in ICU capacity, availability of advanced respiratory support, staffing ratios, and clinical protocols may have influenced both treatment practices and patient outcomes. Finally, the lack of longitudinal laboratory and clinical data limited our ability to evaluate dynamic changes during hospitalization.

These findings highlight the importance of prioritizing high-risk groups—particularly obese and pregnant patients—for early intervention and monitoring. The strong influence of hospital site on mortality underscores the need for equitable distribution of healthcare resources, clinical training, and ICU capabilities across Palestinian healthcare settings.

Future research should aim to validate these findings in larger multicenter cohorts and explore underlying molecular and socioeconomic determinants of observed disparities. Furthermore, incorporating real-time data analytics and risk stratification tools could enhance clinical decision-making and reduce preventable mortality.

This study holds substantial significance in the context of the Palestinian healthcare system. With limited resources, fragmented infrastructure, and recurring public health crises, Palestine faces unique challenges in pandemic response. By identifying specific predictors of mortality within local hospitals, this research provides evidence-based guidance for clinical prioritization, resource allocation, and policy-making. It also emphasizes the need for ongoing investment in health system strengthening and targeted support for vulnerable populations in future public health emergencies.

## Data Availability

The raw data supporting the conclusions of this article will be made available by the authors, without undue reservation.

## References

[1] Ahmed N Khderat AH Sarsour A Taher A Hammoudi A Hamdan Z. The vulnerability of maintenance dialysis patients with COVID-19: mortality and risk factors from a developing country. Ann Med. (2022) 54:1511–1519. 10.1080/07853890.2022.2075914 35594312 PMC9132419

[2] Becerra-Muñoz V Nuñez-Gil I Eid CM Aguado MG Romero R Huang J. Clinical profile and predictors of in-hospital mortality among older patients hospitalised for COVID-19. Age Ageing. 50. (2021). Available online at: https://consensus.app/papers/clinical-profile-and-predictors-of-inhospital-mortality-becerra-mu%C3%B1oz-nu%C3%B1ez-gil/265cf84d4f4d5ad5ab92e5bf45bbd85e/ (Accessed June 15, 2025). 10.1093/ageing/afaa258 PMC7717146 33201181

[3] Dayyab F Bashir HA Sulaiman AK Iliyasu G Hamza M Yakasai A. Determinants of mortality among hospitalized patients with COVID-19 during first and second waves of the pandemic: a retrospective cohort study from an isolation center in Kano, Nigeria. PLoS ONE. (2023) 18:e0281455. 10.1371/journal.pone.0281455 36745658 PMC9901798

[4] Elsorady K Aziz N Daniel S. Predictors of In-Hospital Mortality in Geriatric Patients with COVID-19. (2021). Available online at: https://consensus.app/papers/predictors-of-inhospital-mortality-in-geriatric-patients-elsorady-aziz/cc532b2f4ead5c2f841352afa509c223/ (Accessed June 15, 2025).

[5] Mohammadi Z Dinevari MF Vahed N Bakhtavar HE Rahmani F. Clinical and laboratory predictors of COVID-19-related in-hospital mortality; a cross-sectional study of 1000 cases. Arch Acad Emerg Med. (2022) 10:e49. 10.22037/aaem.v10i1.1574 36033996 PMC9397590

[6] Ozel M Araç S Akkoç H Araç E. Independent predictors of mortality in ICU patients with COVID-19. Dicle Tip Dergisi. (2023) 50:470–81. 10.5798/dicletip.1411504

[7] Banoei M Rafiepoor H Zendehdel K Seyyedsalehi MS Nahvijou A Allameh F. Unraveling complex relationships between COVID-19 risk factors using machine learning based models for predicting mortality of hospitalized patients and identification of high-risk group: a large retrospective study. Front Med. (2023) 10:170331. 10.3389/fmed.2023.1170331 37215714 PMC10192907

[8] Harmouch F Shah K Goel H. 392. Predictors of mortality in hospitalized patients with COVID-19; a single centered retrospective analysis. Open Forum Infect Dis. (2020) 7:S265–S266. 10.1093/ofid/ofaa439.58735928232

[9] Mantha S Jee J Soff G Pessin M. (2020). Prediction of COVID-19 Mortality in Patients with Cancer. Blood, (2020) 136:29–30. 10.1182/blood-2020-138576

[10] Covino M De Matteis G Polla DAD Santoro M Burzo ML Torelli E. Predictors of in-hospital mortality AND death RISK STRATIFICATION among COVID-19 PATIENTS aged ≥ 80 YEARs OLD. Arch Gerontol Geriatr. (2021) 95:104383. 10.1016/j.archger.2021.104383 33676091 PMC7904458

[11] Mandel M Harari G Gurevich M Achiron A. Cytokine prediction of mortality in COVID19 patients. Cytokine. (2020) 134:155190–155190. 10.1016/j.cyto.2020.155190 32673995 PMC7351379

[12] Pietromonaco PR Overall NC. Implications of social isolation, separation, and loss during the COVID-19 pandemic for couples' relationships. Curr Opin Psychol. (2022) 43:189–194. 10.1016/j.copsyc.2021.07.014 34416682 PMC8881098

[13] Raimondi F Novelli L Ghirardi A Russo FM Pellegrini D Biza R. Covid-19 and gender: lower rate but same mortality of severe disease in women—an observational study. BMC Pulmon Med. (2021) 21:96. 10.1183/13993003.congress-2021.PA3663 33743654 PMC7980742

[14] Savoia E Piltch-Loeb R Goldberg B Miller-Idriss C Hughes B Montrond A. Predictors of COVID-19 vaccine hesitancy: socio-demographics, co-morbidity, and past experience of racial discrimination. Vaccines. (2021) 9:767. 10.3390/vaccines9070767 34358184 PMC8310049

[15] Servais T Laurent F Roland T Rossi C Groote ED Godart V. Mortality-related risk factors of inpatients with diabetes and COVID-19: a multicenter retrospective study in Belgium. Ann Endocrinol. (2024) 85:36–43. 10.1016/j.ando.2023.08.002 37574109

[16] Haber R Ghezzawi M Puzantian H Haber M Saad S Ghandour Y. Mortality risk in patients with obesity and COVID-19 infection: a systematic review and meta-analysis. Metabolism: Clin Exp. (2024) 155:155812. 10.1016/j.metabol.2024.155812 38360130

[17] Kurniawati O Prasetya H Murti B. Meta-analysis the effects of obesity and type 2 diabetes mellitus on Covid-19 mortality. J Epidemiol Public Health. (2021) 6:177–91. 10.26911/jepublichealth.2021.06.02.05

[18] Singh R Rathore SS Khan H Karale S Chawla Y Iqbal K. Association of obesity with covid-19 severity and mortality: an updated systemic review, meta-analysis, and meta-regression. Front Endocrinol. (2022). 13:780872. 10.3389/fendo.2022.780872 35721716 PMC9205425

[19] Kim JH Kwon HS Park YM Lee JH Kim MS Yoon KH. Prevalence and associated factors of diabetic retinopathy in rural Korea: the Chungju metabolic disease cohort study. J Korean Med Sci. (2011) 26:1068–1073. 10.3346/jkms.2011.26.8.1068 21860558 PMC3154343

[20] Venkat DG Badr M. Respiratory disorders and their association with clinical outcomes in COVID-19: a narrative review of current literature. Ann Palliat Med. (2023) 12:1232–1243. 10.21037/apm-22-1427 37164968

[21] Torres-Torres J Martínez-Portilla R Espino-y-Sosa S Estrada-Gutiérrez G Solis-Paredes J Villafán-Bernal J. Comorbidity, poverty and social vulnerability as risk factors for mortality in pregnant women with confirmed SARS-CoV-2 infection: Analysis of 13 062 positive pregnancies including 176 maternal deaths in Mexico. Ultra Obstetr Gynecol. (2021) 59, 76–82. 10.1002/uog.24797 34672382 PMC8662032

[22] Zamparini J Saggers R Buga CE. A review of Coronavirus Disease 2019 in pregnancy. Semin Respir Crit Care Med. (2023) 44:50–065. 10.1055/s-0042-1758853 36646085

[23] Bshaena A Almajdoub OH Alshwesh R Omran E Haq S Ismail F. (2022). Association between ABO blood group system and COVID-19 severity. Am J Clin Pathol. 158:570–573. 10.1093/ajcp/aqac106 36069364 PMC9494408

[24] Ishaq U Malik A Malik J Mehmood A Qureshi A Laique T.. Association of ABO blood group with COVID-19 severity, acute phase reactants and mortality. PLoS ONE. (2021) 16:e0261432. 10.1371/journal.pone.0261432 34905588 PMC8670663

[25] Churpek M Gupta S Spicer A Parker W Fahrenbach J Brenner SK. Hospital-level variation in death for critically Ill patients with COVID-19. Am J Respir Crit Care Med. (2021) 204:403–411. 10.1164/rccm.202012-4547OC 33891529 PMC8480242

[26] Fomin VV Royuk VV Reshetnikov V Volkova OS Korshever NG Kozlov VV. Analysis of In-Hospital Mortality of Patients with New Coronavirus Infection (COVID-19) of Clinical Centre of Sechenov University. IP Pavlov Russian Medical Biological Herald. (2023) 31: 381–389. 10.17816/PAVLOVJ569334

[27] Ebrahimi V Sharifi M Mousavi-roknabadi RS Sadegh R Khademian M Moghadami M. Predictive determinants of overall survival among re-infected COVID-19 patients using the elastic-net regularized Cox proportional hazards model: A machine-learning algorithm. BMC Public Health. (2021) 22:10. 10.1186/s12889-021-12383-3 34986818 PMC8727465

[28] Ghazanfari T Salehi M Namaki S Arabkheradmand J Rostamian A Chenary MR. Interpretation of hematological, biochemical, and immunological findings of COVID-19 disease: biomarkers associated with severity and mortality. Iran J Allergy Asthma Immunol. (2021) 20:46–66. 10.18502/ijaai.v20i1.5412 33639632

[29] Shreewastav RK Singh G Jha KK Yadav R Gharti SB. Study on status of serum biochemical and hematological parameters in COVID-19 positive patients attending a tertiary care hospital. Kathman Univ Med J. (2022) 20: 295–300. 10.3126/kumj.v20i3.53934 37042369

[30] Bhowmik KK Barek MA Aziz MA Islam MS. (2022). A systematic review and meta-analysis of abnormalities in hematological and biochemical markers among Bangladeshi COVID-19 cases. Health Sci. Rep. 5:e728. 10.1002/hsr2.728 35899180 PMC9309618

[31] Srivastava A Bhardwaj A. prognostic role of nlr and rbs levels in diabetic patients with severe. Covid-19 disease. Int J Sci Res. (2021). 10.36106/ijsr/7200107

[32] Tharakan S Nomoto K Miyashita S Ishikawa K. Body temperature correlates with mortality in COVID-19 patients. Crit. Care. (2020). 24:298. 10.1186/s13054-020-03045-8 32503659 PMC7274509

[33] Uchiyama S Sakata T Tharakan S Ishikawa K. Body temperature as a predictor of mortality in COVID-19. Sci. Rep. (2023) 13:13354. 10.1038/s41598-023-40414-z 37587219 PMC10432378

[34] Gokhale Y Mehta R Kulkarni U Karnik N Gokhale S Sundar U. Tocilizumab improves survival in severe COVID-19 pneumonia with persistent hypoxia: a retrospective cohort study with follow-up from Mumbai, India. BMC Infect Dis. (2021). 10.21203/rs.3.rs-80152/v2 33673818 PMC7934984

[35] Kyriakopoulos C Ntritsos G Gogali A Milionis H Evangelou E Kostikas K. Systematic review and meta-analysis of tocilizumab administration for the treatment of hospitalised patients with Covid-19. SSRN Electron J. (2021). 37. 10.2139/ssrn.3844920 PMC8661720 34605114

[36] Tolzali MMR Noori M Shokri P Rahmani S Khanzadeh S Nejadghaderi SA. Efficacy of tocilizumab in the treatment of COVID-19: an umbrella review. Rev Med Virol. (2022) 32:e2388. 10.1002/rmv.2388 36029180 PMC9539231

[37] Song JW Chung KC. Observational studies: cohort and case-control studies. Plast Reconstr Surg. (2010) 126:2234–42. 10.1097/PRS.0b013e3181f44abc 20697313 PMC2998589

[38] World Health Organization (2025). Global COVID-19 Pandemic Situation Report. World Health Organization. Available online at: https://www.who.int

